# Remote Assessment of Disease and Relapse in Major Depressive Disorder (RADAR-MDD): recruitment, retention, and data availability in a longitudinal remote measurement study

**DOI:** 10.1186/s12888-022-03753-1

**Published:** 2022-02-21

**Authors:** Faith Matcham, Daniel Leightley, Sara Siddi, Femke Lamers, Katie M. White, Peter Annas, Giovanni de Girolamo, Sonia Difrancesco, Josep Maria Haro, Melany Horsfall, Alina Ivan, Grace Lavelle, Qingqin Li, Federica Lombardini, David C. Mohr, Vaibhav A. Narayan, Carolin Oetzmann, Brenda W. J. H. Penninx, Stuart Bruce, Raluca Nica, Sara K. Simblett, Til Wykes, Jens Christian Brasen, Inez Myin-Germeys, Aki Rintala, Pauline Conde, Richard J. B. Dobson, Amos A. Folarin, Callum Stewart, Yatharth Ranjan, Zulqarnain Rashid, Nick Cummins, Nikolay V. Manyakov, Srinivasan Vairavan, Matthew Hotopf, on behalf of the RADAR-CNS consortium

**Affiliations:** 1 Institute of Psychiatry, Psychology and Neuroscience, King’s College London, London, UK; 2 Parc Sanitari Sant Joan de Déu, Fundació Sant Joan de Déu, CIBERSAM, Universitat de Barcelona, Barcelona, Spain; 3 Department of Psychiatry and Amsterdam Public Health Research Institute, Amsterdam UMC, Vrije Universiteit, Amsterdam, The Netherlands; 4 H. Lundbeck A/S, Valby, Denmark; 5 IRCCS Instituto Centro San Giovanni di Dio Fatebenefratelli, Brescia, Italy; 6 Janssen Research and Development, LLC, Titusville, NJ USA; 7 Center for Behavioral Intervention Technologies, Department of Preventative Medicine, Northwestern University, Chicago, IL USA; 8 RADAR-CNS Patient Advisory Board, King’s College London, London, UK; 9 Department for Neurosciences, Center for Contextual Psychiatry, KU Leuven, Leuven, Belgium; 10 Faculty of Social and Health Care, LAB University of Applied Sciences, Lahti, Finland; 11 Department of Biostatistics and Health Informatics, Institute of Psychiatry, Psychology and Neuroscience, King’s College London, London, UK; 12 Chair of Embedded Intelligence for Health Care and Wellbeing, University of Augsburg, Augsburg, Germany; 13 Janssen Pharmaceutica NV, Beerse, Belgium; 14 South London and Maudsley NHS Foundation Trust, London, UK; 15 https://www.radar-cns.org

**Keywords:** Major depressive disorder, Remote measurement technologies, Longitudinal, Multicentre, Cohort study

## Abstract

**Background:**

Major Depressive Disorder (MDD) is prevalent, often chronic, and requires ongoing monitoring of symptoms to track response to treatment and identify early indicators of relapse. Remote Measurement Technologies (RMT) provide an opportunity to transform the measurement and management of MDD, via data collected from inbuilt smartphone sensors and wearable devices alongside app-based questionnaires and tasks. A key question for the field is the extent to which participants can adhere to research protocols and the completeness of data collected. We aimed to describe drop out and data completeness in a naturalistic multimodal longitudinal RMT study, in people with a history of recurrent MDD. We further aimed to determine whether those experiencing a depressive relapse at baseline contributed less complete data.

**Methods:**

Remote Assessment of Disease and Relapse – Major Depressive Disorder (RADAR-MDD) is a multi-centre, prospective observational cohort study conducted as part of the Remote Assessment of Disease and Relapse – Central Nervous System (RADAR-CNS) program. People with a history of MDD were provided with a wrist-worn wearable device, and smartphone apps designed to: a) collect data from smartphone sensors; and b) deliver questionnaires, speech tasks, and cognitive assessments. Participants were followed-up for a minimum of 11 months and maximum of 24 months.

**Results:**

Individuals with a history of MDD (*n* = 623) were enrolled in the study,. We report 80% completion rates for primary outcome assessments across all follow-up timepoints. 79.8% of people participated for the maximum amount of time available and 20.2% withdrew prematurely. We found no evidence of an association between the severity of depression symptoms at baseline and the availability of data. In total, 110 participants had > 50% data available across all data types.

**Conclusions:**

RADAR-MDD is the largest multimodal RMT study in the field of mental health. Here, we have shown that collecting RMT data from a clinical population is feasible. We found comparable levels of data availability in active and passive forms of data collection, demonstrating that both are feasible in this patient group.

**Supplementary Information:**

The online version contains supplementary material available at 10.1186/s12888-022-03753-1.

## Background

Globally, depressive disorders contribute to 14.3% of all-age years lived with disability (YLD), making it the third leading cause of YLD [1]. Major depressive disorder (MDD) is a severe form of depression characterised by prolonged periods of low mood and anhedonia combined with a range of other symptoms including changes in sleep quality, appetite, cognitive function, energy levels, activity, feelings of guilt or worthlessness and thoughts of death [2]. MDD is associated with a wide range of negative outcomes including: loss of occupational function [3], reduced quality-of-life [4], and premature mortality [5]. Whilst some may experience a single lifetime episode of MDD, it is becoming more widely recognised as a chronic condition, characterised by periods of relapse and recovery [6, 7]. The management of chronic illnesses requires ongoing monitoring of symptoms, for example to track response to treatment or identify early indicators of relapse. This monitoring is dependent on self-reported questionnaires or clinical interviews, which are typically infrequent (e.g. conducted at clinic visits) and reliant on individuals’ recollection of symptoms, and subject to recall bias [8].

The use and ownership of smartphones and wearable technology has increased exponentially in the last decade. These technologies provide the opportunity to collect data using unobtrusive, inbuilt sensors requiring minimal input from users [9, 10]. In additional to unobtrusive passive data collection, there is scope for more frequent self-report information to be collected. Many features of MDD are amenable to assessment via remote measurement technologies (RMT): for example, heart rate from photoplethysmography (PPG) sensors and activity from accelerometery sensors in wrist-worn wearable devices can give information indicative of sleep patterns and physical activity levels. Data such as Global Positioning System (GPS), Bluetooth, gyroscope, phone screen interactions, ambient noise and light levels have also been used to collect information from smartphones relating to sociability, movement and activity associated with low mood [11]. In contrast to this passive RMT (pRMT) form of data collection, which requires little or no input from the user, active RMT (aRMT), deliverable by smartphone, requires the user to respond to a notification and complete, for example, short questionnaires, cognitive tasks or speech sampling tasks. Combining these active and passive data streams could potentially provide a real-time overview of the patient’s health status which could inform treatment delivery. It could further be used to predict future changes in health states – for example signals might be identified to predict a relapse in an otherwise healthy individual [12]. A key question in the use of smartphones and wearables to track health is that these technologies require considerable commitment from participants and/or patients. Not only must they consent for their personal smartphone data to be used, they must also be motivated to wear wrist-worn devices, to maintain such devices (e.g. to have them charged) and to interact with their phones to provide active RMT data. Whilst the wider field of digital medicine has seen vast growth and investment, many technologies have poor uptake [13, 14]. In depression the illness, characterised by loss of motivation, may be a further barrier to adherence with digital medicine protocols [15, 16]. If such technologies are to be used in real-world settings they therefore have to have high acceptability. A key question for the field is therefore the extent to which people with depression will adhere to such protocols. In a recent systematic review we identified 52 publications testing RMT in depression [17]. The literature was characterised by inconsistent reporting, and very rarely were data on adherence to protocol reported.

The study reported here, Remote Assessment of Disease and Relapse in Major Depressive Disorder (RADAR-MDD) [18], is a longitudinal cohort study examining the utility of multi-parametric RMT to measure changes in symptoms and predict relapse in people with MDD. The study was designed with patient involvement from the outset (including systematic reviews [19, 20], focus groups [21] and a Patient Advisory Board) with the aim of developing a protocol which meets the needs of the target population. RADAR-MDD offers an opportunity to explore the recruitment of people with MDD into a complex digital technology study, and describe the long-term retention rates and adherence to a protocol which includes passive data collection via smartphone and wearable sensors, app-based questionnaires, experience sampling method (ESM) and traditional web-based outcome assessments [18].

Throughout this paper, we have used the term data “availability” instead of “completeness” as we describe all data provided throughout the study, regardless of quality or completeness. Data labelled as “available” in this paper may include i) complete, valid data which are usable for analysis; ii) partial data which are incomplete but potentially usable; and iii) data which have been corrupted or are invalid for any reason. We believe it is essential to include partial or incomplete data as part of this paper, as they are indicative not only of participant characteristics and study burden, but also of the underlying technical infrastructure. We decided to not withdraw participants for not providing data via the smartphone apps or wearable devices. This concession gives greater insight into how data availability may fluctuate with changes in depressive state and provides a truer representation of the feasibility of implementing RMT protocols in people with MDD.

The aims of this paper are to: 1) summarise study recruitment, retention, and completion rates of primary and secondary participant-reported outcomes throughout the course of follow-up; 2) describe the sociodemographic and clinical characteristics of the cohort for the RADAR-MDD study; 3) describe the availability of data throughout a multi-parametric RMT study protocol including active and passive assessments of symptoms, behaviour and cognitive function and 4) determine whether participants with depression at baseline had poorer data availability.

## Methods

### Study design

The full protocol for RADAR-MDD has been reported elsewhere [18]. In short, RADAR-MDD is a multi-centre, prospective observational cohort study. The study aimed to examine whether data collected via multiparametric RMT can be used to reliably track illness course and predict relapse in MDD. The study sought to recruit 600 individuals with a recent history of recurrent MDD (with the latest episode within the past 2 years) and follow them up for a maximum of 24 months. The study has three recruitment sites: King’s College London (KCL, UK), Amsterdam University Medical Centre (VUmc. Amsterdam, The Netherlands), and Centro de Investigación Biomédica en Red (CIBER; Barcelona, Spain).

### Study population

To be eligible for participation in RADAR-MDD, individuals must: 1) have met DSM-5 diagnostic criteria for non-psychotic MDD within the past 2 years; 2) have recurrent MDD (having had a lifetime history of at least 2 episodes); 3) be able and willing to complete self-reported assessments via smartphone; 4) be able to give informed consent; 5) be fluent in English, Dutch, Spanish or Catalan; 5) have an existing Android smartphone, or willingness to swap to Android as their only phone; 6) be aged 18 or over. Depression diagnosis was determined using the Lifetime Depression Assessment – Self-Report (LIDAS; [22]) in addition to the review of medical records.

Exclusion criteria were: 1) having a self-reported lifetime history of bipolar disorder, schizophrenia, MDD with psychotic features, or schizoaffective disorder; 2) dementia; 3) having received treatment for drug or alcohol use in the 6 months prior to enrolment; 4) a major medical diagnosis which might impact an individual’s ability to participate in normal daily activities for more than 2 weeks; 5) pregnancy (although once enrolled, becoming pregnant did not result in withdrawal as pre-pregnancy baseline data had already been obtained).

Eligible participants were identified via several recruitment channels, including through existing research cohorts who have consented to be contacted for future research opportunities (in the UK [23] and the Netherlands), through primary and secondary mental health services (in the UK and Barcelona), or through advertisements for the study placed on mental health charity websites, circulars or Twitter notices (at all sites). Participants in Amsterdam were partially recruited through Hersenonderzoek.nl (https://hersenonderzoek.nl/). All participants provided written consent and provided detailed baseline assessments including sociodemographic, social environment, medical history, medical comorbidities and technology use questionnaires.

#### Data collection

##### Remote data collection

Data collection started in London (UK) in November 2017 in a pilot phase of app development, with additional assessments being added to the protocol throughout the first 18 months of the study period to allow small-scale functionality testing and quality control before international large-scale data collection commenced. Data collection started in Barcelona and Amsterdam in September 2018 and February 2019, respectively. The data collected used RADAR-base, an open-source platform designed to leverage data from wearables and mobile technologies [24]. RADAR-base provides both passive and active data collection via two apps – the RADAR active and passive monitoring apps.

#### Passive RMT

The passive RMT (pRMT) app unobtrusively collected information about phone usage throughout participation, requiring no input from the participant. It collected data on ambient noise, ambient light, location, app usage, Bluetooth connectivity, phone usage, and battery life. Some data sources were removed from the protocol throughout follow-up (summarised in Supplementary file 1) due to unavoidable changes in smartphone operating systems. Changes to Google’s Play Store permissions prevented access to text and call log data as of January 2019. Data pertaining to text and call logs have not been reported in the current paper due to data collection from this sensor ceasing when one site had only recruited 30 individuals and another site had not started recruitment at all. Participants were additionally asked to wear a Fitbit Charge 2/3 device for the duration of participation, providing information about individuals’ sleep and physical activity levels. Participants could keep the Fitbit at the end of the time in the study.

#### Active RMT

The RADAR-base active RMT (aRMT) app administered validated measurements of depression and self-esteem every 2-weeks via the 8-item Patient Health Questionnaire (PHQ8; [25] and Rosenberg Self-Esteem Scale (RSES; 26). Items on the PHQ8 can be totalled and used as a continuous score with higher scores indicating increased depression severity, and scores totalling ≥10 indicating those with significant symptoms [25]. The RSES requires reversing of 5 of the 10 items, which then can be totalled to create a total score with higher scores representing increased self-esteem [26].

The aRMT app also delivered a speech task every 2-weeks, requesting participants to record a pre-determined text from the “North Wind and the Sun” (see Supplementary file 2), an Aesop’s fable which is phonetically balanced across all three languages and has been shown to provide linguistic parameters indicative of low mood [27]. Participants were also asked to provide a sample of speech in answer a question relating to plans for the upcoming week. Finally, the aRMT app included an ESM protocol [18], requiring participants to complete brief questions relating to mood, stress, sociability, activity and sleep, multiple times per day for 6 days at scheduled times throughout the course of follow-up.

#### Cognitive function

Cognitive function was measured every 6-weeks via an additional THINC-it app®, which was integrated into the RADAR-base platform. The app has been validated to identify cognitive dysfunction within the context of depressive disorder [28]. The app contains the 5-item Perceived Deficits Questionnaire (PDQ-5; [29]), alongside computerised versions of the Choice Reaction Time Identification Task (“Code Breaker”), One-Back Test (“Spotter”), Digit Symbol Substitution Test (“Symbol Check”) and Trail Making Test-Part B (“Trails”) tasks to assess processing speed, working memory, concentration and attention [28].

##### Primary and secondary outcome assessments

All primary and secondary outcome measurements were collected via automatic surveys sent every 3 months via the Research Electronic Data Capture (REDCap) software [30]. A full description of the outcome assessment schedule is provided in our published protocol paper [18].

#### Depression

Depressive state was measured using the Inventory of Depressive Symptomatology – Self Report (IDS-SR; [31]) to capture changes in symptom severity, and the World Health Organisation’s Composite Diagnostic Interview – Short Form (CIDI-SF; [32]) to identify people meeting DSM-5 criteria for MDD at each timepoint. These two measurements were used to identify different operationalisations of depression across follow-up, summarised in Supplementary file 3. Briefly, participants were categorised as being “symptomatic” (scoring ≥26 on the IDS-SR and meeting CIDI-SF criteria for MDD), having “some symptoms” (scoring ≤25 on the IDS-SR and meeting CIDI-SF criteria for MDD; or > 21 on the IDS-SR and not meeting CIDI-SF criteria for MDD) or having “no/mild symptoms” (scoring ≤21 on the IDS-SR and not meeting CIDI-SF criteria for MDD).

As described previously [18], the primary outcome of interest in RADAR-MDD is depressive relapse, defined here as switching from a state of “no/mild symptoms” to “symptomatic” over a period of 6-months. Secondary depression outcomes are: remission (switching from a state of “symptomatic” to “no/mild symptoms” over a period of 6-months); and change in the severity of depressive symptoms (measured via the continuous IDS-SR).

#### Anxiety

Anxiety was measured via the 7-item Generalised Anxiety Disorder questionnaire (GAD7; [33]), used as a continuous indicator of anxiety symptom severity (a total of 21, with higher scores indicating increased anxiety severity) and a total score ≥ 10 indicating significant symptoms. This threshold has previously been shown to have good levels of sensitivity and specificity [34].

#### Functional ability

Functional ability was measured using the Work and Social Adjustment Scale (WSAS; [35]), using a continuous score from 0 to 40 to describe the level of impairment, with scores of 0–10, 11–20 and > 20 to indicate no, some and significant impairment respectively [35].

#### Alcohol use

The Alcohol Use Disorders Identification Test (AUDIT; [36]) was used to measure alcohol use across timepoints. A total score out of 40 describes the level of alcohol use; scores of 0–7 indicate low risk alcohol consumption; 8–15 indicate hazardous alcohol consumption; 16–19 indicate harmful alcohol consumption; and scores > 20 indicate likely alcohol dependence [37].

#### Illness perceptions

The Brief Illness Perceptions Questionnaire (BIPQ; [38]) measured emotional and cognitive representations of illness, capturing perceptions relating to illness identity, causes, control, consequences, timeline, concern, understanding and emotional response. Total scores for each domain can be used individually, or totalled, with higher scores representing a more threatening view of their illness.

#### Health service use

Access to health services, as well as changes in treatment, and care received was measured via a modified Client Service Receipt Inventory (CSRI; [39]), adapted to be suitable for online delivery and participant self-report.

### Covariates

#### Life events

Any significant life events which may have happened between outcome assessments were measured via the List of Threatening Experiences Questionnaire (LTE-Q; [40]). Changes in employment status were recorded regularly as part of the CSRI [39].

#### Medication adherence

Self-reported adherence to depression medication was measured with the 5-item Medication Adherence Report Scale (MARS-5; [41]).

#### Patient and Public Involvement

The study was co-developed with service users in our Patient Advisory Board. They were involved in the choice of measures, the timing and issues of engagement and have also been involved in developing the analysis plan and representative(s) are authors of this paper and critically reviewed it.

#### Statistical analyses

Baseline characteristics of the sample were described using means and standard deviations or numbers and percentages as appropriate. To examine whether depressed mood is associated with the availability of data across all modes of data collection, participants were divided using scores on the IDS-SR and CIDI-SF (see Supplementary file 3 for operationalisation) into those who are symptomatic at baseline and those who are not (those with no/mild symptoms and some symptoms are pooled together due to the low number of people with no/mild symptoms at baseline (*n* = 4)). Chi-squared tests examined differences between those with baseline symptoms of depression and those without in categorial data, and linear regressions in continuous data.

The number and percentage of people who have provided any data via the aRMT and pRMT apps and the wearable device throughout the course of follow-up have been summarised, then divided into quartiles to examine the numbers of people who have provided 0–25% of expected data, 26–50%, 51–75 and > 75% of data throughout follow-up. Fitbit wear time estimates were calculated based on the presence of a single heart rate value, greater than zero, per 15-min window.

*P*-values comparing the amount of data available between people with symptoms of depression at baseline and those without symptoms of depression at baseline were created using Chi-Squared tests. T-tests compared the number of ESM questions completed in total across all follow-up timepoints between those with and without depression symptoms at baseline. Data were analysed using STATA v16.0.

## Results

### Recruitment and retention rates

**Fig. 1:**
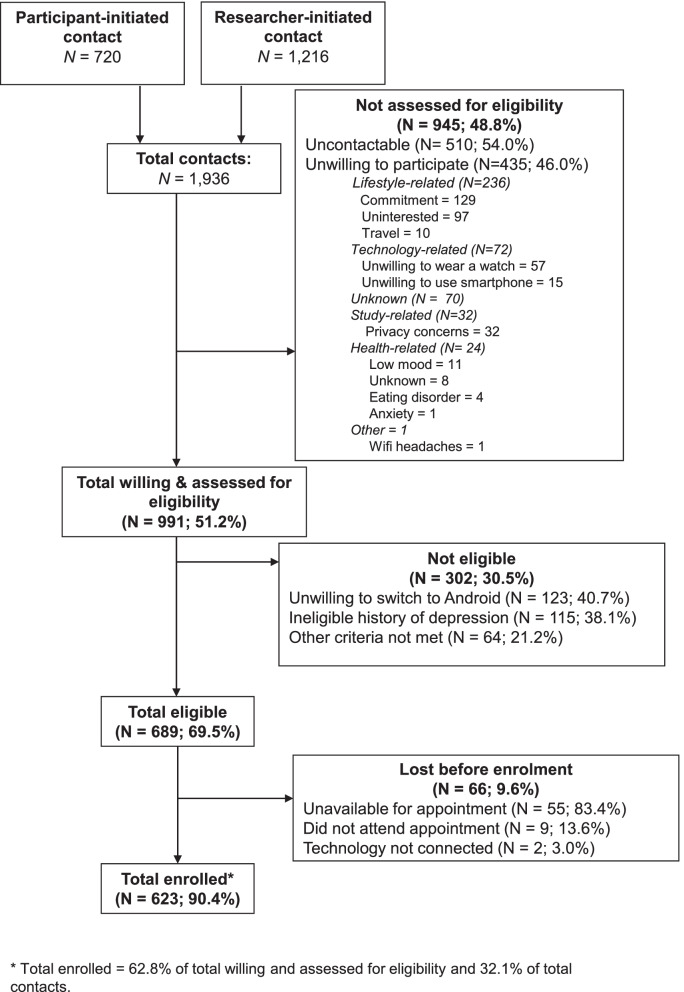
STROBE flowchart for recruitment into RADAR-MDD

The first person was enrolled in RADAR-MDD on 30th November 2017, and recruitment ended on 3rd June 2020, representing a total of 30 months of recruitment. Figure 1 shows the participation rate, detailing the total number of participants contacted and the reasons for non-participation.

**Fig. 2:**
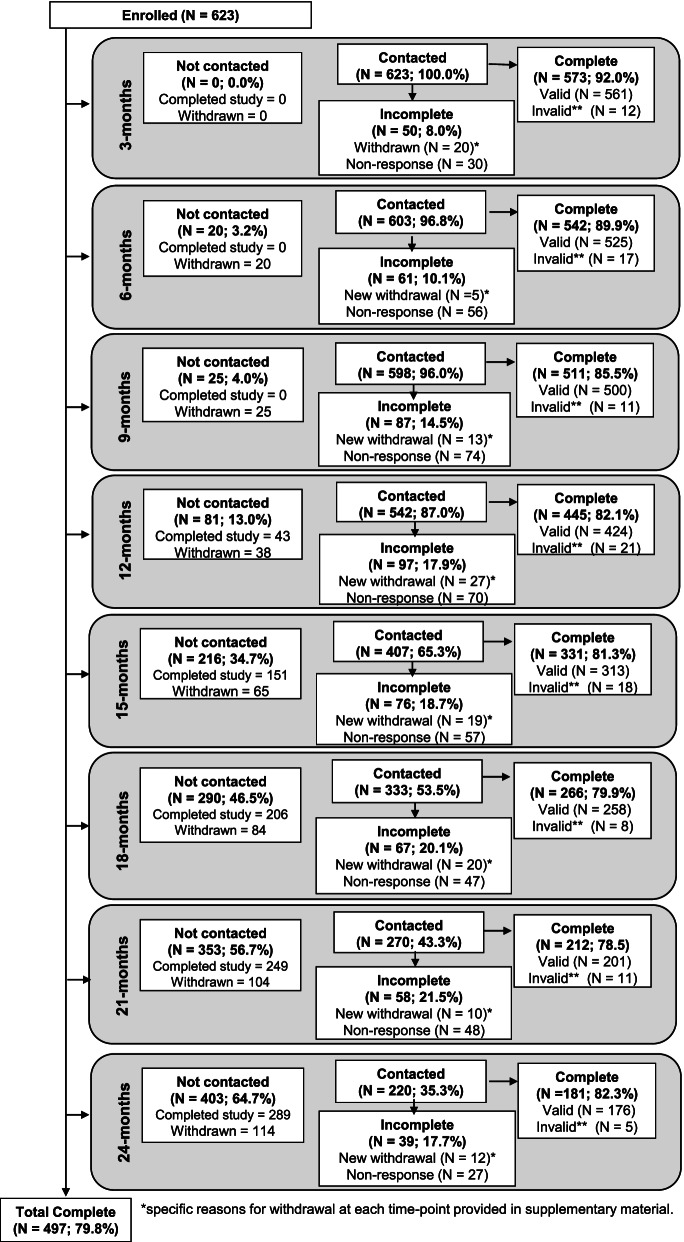
Participants “not contacted” because they had already completed the maximum amount of follow-up time or had already withdrawn from the study. Participants were “contacted” when they were still active participants. *Reasons for withdrawal provided in Supplementary file 4. **Invalid outcomes collected ±21 days of due date

Figure 2 shows the participant retention rate throughout the period of follow-up. At each timepoint, the number of people eligible for contact for an outcome assessment decreased as: 1) more people had reached the end of the data collection period; and 2) as people had been withdrawn from the study. As the last participant was recruited in June 2020 and the study finished in April 2021, the minimum and maximum lengths of possible follow-up were 11 months and 24 months respectively. The completion rate of the primary and secondary outcomes in those who were eligible to complete it (those who had not already completed the study or been previously withdrawn) was approximately 80% throughout follow-up assessments.

Of the 623 participants enrolled in the study, 445 (71.4%) provided outcome data at 1-year follow-up and 181 (29.1%) participated for a full 2-years. A total of 497 people (79.8%) participated for the maximum possible duration (from their enrolment until the end of data collection in April 2021), and 126 people (20.2%) withdrew prematurely. Reasons for withdrawal are provided in Supplementary file 4. The most common reason for withdrawal across all timepoints was loss to follow-up (*n* = 47) and problems using the Android study phone (for those who had switched from an iPhone for the purposes of the study (*n* = 14), representing 37.3 and 11.1% of all withdrawals respectively. A total of 8 participants identified study burden as the main reason for withdrawal, including finding the study “too demanding” (*n* = 6) or the study “not meeting expectations” (*n* = 2).

#### Sample characteristics

The target sample size of 600, across the three sites, was exceeded, with 623 individuals successfully enrolled in the study. The baseline sociodemographic and clinical characteristics of this sample are displayed in Table 1, with comparisons made between those with no/some symptoms at baseline and those who were symptomatic at baseline (see Supplementary file 5 for between-site stratification).

**Table 1:** Sociodemographic and clinical baseline data and comparisons between those with no/some depression symptoms at baseline, and those who are symptomatic at baseline

		Total Sample	No/Some Baseline Depression Symptoms^**a**^ (***n*** = 245)	High Baseline Depression Symptoms (***n*** = 378)^**b**^	***P***-value
Total, N(%)		623 (100.0)	245 (39.3)	378 (60.7)	
London, N(%)		350 (56.2)	149 (60.8)	201 (53.2)	0.005
Barcelona, N(%)		155 (24.9)	44 (18.0)	111 (29.4)	
Amsterdam, N(%)		118 (18.9)	52 (21.2)	66 (17.5)	
*Socio-demographics*					
Age, M(SD)		46.4 (15.3)	48.2 (15.4)	45.1 (15.0)	0.013
Gender, N(%)	Female	471 (75.6)	171 (69.8)	300 (79.4)	0.007
Marital Status, N(%)	Single/separated/divorced/widowed	332 (53.3)	119 (47.8)	213 (56.2)	0.070
	Married/cohabiting/LTR	291 (46.7)	125 (51.2)	166 (43.8)	
Aggregated Ethnicity, N(%)*	White British/Dutch	369 (78.9)	163 (81.1)	206 (77.2)	0.262
	White Other	35 (7.5)	18 (9.0)	17 (6.4)	
	Black ethnic group	14 (3.0)	3 (1.5)	11 (4.1)	
	Asian ethnic group	16 (3.4)	7 (3.5)	9 (3.4)	
	Mixed ethnic background	16 (3.4)	5 (2.5)	11 (4.1)	
	Other	18 (3.9)	5 (2.5)	13 (4.9)	
Employment Status	Employed/furloughed	260 (41.7)	120 (49.2)	140 (36.9)	< 0.0001
	Unemployed/sick leave	134 (21.5)	35 (14.3)	99 (26.1)	
	Student	68 (10.9)	21 (8.6)	47 (12.4)	
	Retired	123 (19.7)	58 (23.8)	65 (17.2)	
	Not reported	38 (6.1)	10 (4.1)	28 (7.4)	
Total years in education, M(SD)		16.4 (6.5)	17.0 (6.7)	16.1 (6.3)	0.085
Benefits Receipt, N(%)	Yes	275 (44.1)	91 (37.1)	184 (48.7)	0.005
Accommodation type, N(%)	Own outright/with mortgage	368 (59.1)	150 (61.5)	218 (57.5)	0.425
	Renting	216 (34.7)	83 (34.0)	133 (35.1)	
	Living rent-free	29 (4.7)	9 (3.7)	20 (5.3)	
	Not reported	10 (1.6)	2 (0.8)	8 (2.1)	
Household income per annum, N(%)	<£/€15,000	154 (24.8)	43 (17.6)	111 (29.4)	0.003
	£/€15,000 – 55,000	354 (57.0)	143 (58.4)	211 (55.8)	
	>£€55,000	98 (15.8)	52 (21.2)	46 (12.2)	
	Prefer not to say	10 (1.6)	3 (1.2)	7 (1.9)	
	Unknown	5 (0.8)	3 (1.2)	2 (0.5)	
*Clinical Characteristics*					
Current smoker, N(%)	Yes	126 (20.2)	38 (15.5)	88 (23.3)	0.014
Medical comorbidity, N(%)	Yes	343 (55.1)	111 (45.3)	232 (61.4)	< 0.0001
Lifetime traumatic events, N(%)	None	66 (10.6)	28 (11.4)	38 (10.1)	0.440
	1–5	360 (57.8)	149 (60.8)	212 (56.1)	
	6–12	185 (29.7)	65 (26.5)	121 (32.0)	
	Not reported	12 (1.9)	3 (1.2)	7 (1.9)	
Current depression	IDS-SR total, M(SD)	31.3 (14.5)	17.5 (8.3)	39.7 (10.5)	< 0.0001
	None (0–13), N(%)	61 (10.1)	61 (24.9)	0 (0.0)	< 0.0001
	Mild (14–25), N(%)	157 (25.9)	157 (64.1)	0 (0.0)	
	Moderate (26–38), N(%)	206 (33.9)	4 (1.6)	202 (53.4)	
	Severe (39–48), N(%)	104 (17.1)	5 (2.0)	99 (26.2)	
	Very severe (49–84), N(%)	79 (13.0)	2 (0.8)	77 (20.4)	
	Not reported	16 (2.6)	16 (6.5)	0 (0.0)	
Suicidal ideation, N(%)	Yes	110 (17.7)	13 (5.3)	97 (25.7)	< 0.0001
Taking antidepressants, N(%)	Yes	408 (65.5)	142 (58.0)	266 (70.4)	0.004
Current anxiety	GAD7 total, M(SD)	8.8 (5.7)	5.3 (4.2)	11.0 (5.0)	< 0.0001
	≥10, N(%)	270 (43.3)	46 (18.9)	224 (59.1)	< 0.0001
Current functional disability	WSAS total, M(SD)	19.3 (11.1)	12.0 (9.9)	23.9 (9.1)	< 0.0001
	No impairment (0–10), N(%)	155 (24.9)	126 (51.4)	29 (7.7)	< 0.0001
	Some impairment (11–20), N(%)	154 (24.7)	57 (23.3)	97 (25.7)	
	Significant impairment (> 20), N(%)	314 (50.4)	62 (25.3)	252 (66.7)	
Alcohol use	AUDIT total, M(SD)	3.2 (4.4)	3.9 (4.6)	2.8 (4.3)	0.005
	Low risk (0–7), N(%)	528 (84.8)	198 (80.8)	330 (87.3)	0.242
	Medium risk (8–15), N(%)	52 (8.4)	25 (10.2)	27 (7.1)	
	High risk (16–19), N(%)	10 (1.6)	5 (2.0)	5 (1.3)	
	Addiction likely (> 19), N(%)	8 (1.3)	5 (2.0)	3 (0.8)	
	Not reported	25 (4.0)	12 (4.9)	13 (3.4)	
Illness Perceptions, M(SD)	Consequences	6.1 (2.8)	4.5 (2.8)	7.1 (2.3)	< 0.0001
	Timeline	7.1 (3.1)	5.8 (3.6)	7.9 (2.4)	< 0.0001
	Personal Control	4.2 (2.7)	4.8 (2.7)	3.8 (2.6)	< 0.0001
	Treatment Control	6.0 (2.8)	6.7 (2.9)	5.5 (2.6)	< 0.0001
	Identity	5.9 (2.5)	4.5 (2.6)	6.7 (2.0)	< 0.0001
	Concern	6.3 (2.9)	4.9 (3.0)	7.2 (2.5)	< 0.0001
	Understanding	6.8 (2.8)	7.2 (2.7)	6.6 (2.9)	0.012
	Emotional Response	7.1 (2.5)	6.1 (2.9)	7.9 (2.0)	< 0.0001
Baseline aRMT PHQ8	PHQ8 total, M(SD)	10.9 (6.0)	6.4 (4.6)	13.7 (5.0)	< 0.0001
	≥10, N(%)	371 (59.6)	69 (28.3)	302 (79.7)	< 0.0001
Baseline aRMT RSES (*N* = 545)	RSES total, M(SD)	36.8 (2.3)	36.7 (2.4)	36.9 (2.3)	0.277

^a^ total number of participants not indicated as symptomatic. ^b^ total number of symptomatic: participants meeting criteria for MDD on the CIDI-SF and scoring > 25 on the IDS-SR. LTR Long Term Relationship. *Ethnicity data not collected at Spanish site (*N* = 155), percentages reported out of 468 individuals. Ethnicity data aggregated according to recommendations provided here: https://www.ethnicity-facts-figures.service.gov.uk/style-guide/writing-about-ethnicity. IDS-SR Inventory of Depressive Symptomatology – Self Report. GAD7 7-item questionnaire for Generalised Anxiety Disorder. WSAS Work and Social Adjustment Scale. AUDIT Alcohol Use Disorders Identification Test. BIPQ Brief Illness Perceptions Questionnaire. PHQ8 8-item Patient Health Questionnaire. RSES Rosenberg Self-Esteem Scale. M(SD) Mean (Standard Deviation)

In comparison to those with no/some depression symptoms at baseline, the symptomatic group were significantly younger, and had a higher proportion of individuals who were female, on long-term sick leave or unemployed, receiving benefits, and earning less than £/€15,000 per annum. Regarding clinical characteristics, the symptomatic group had a higher proportion of current smokers, medical comorbidities, as well as increased levels of current depression, anxiety, functional disability, and worsened illness perceptions, although lower levels of alcohol use. Throughout RADAR-MDD, a total of 341 risk assessments were conducted (9.0% of the 3777 depression measurements taken).

#### Data collection with RMT

Data collection started on 30th November 2017, with data collection continuing until the last participant was unenrolled from the study on 1st May 2021, resulting in a median study duration of participation of 541 days (interquartile range (IQR): 401–730 days, range: 0–1217 days). A total of 2.9 terabytes of compressed data were collected, with 110 (17.7%) participants having more than 50% available data across all modes of data collection.

##### Data collected via aRMT

**Fig. 3:**
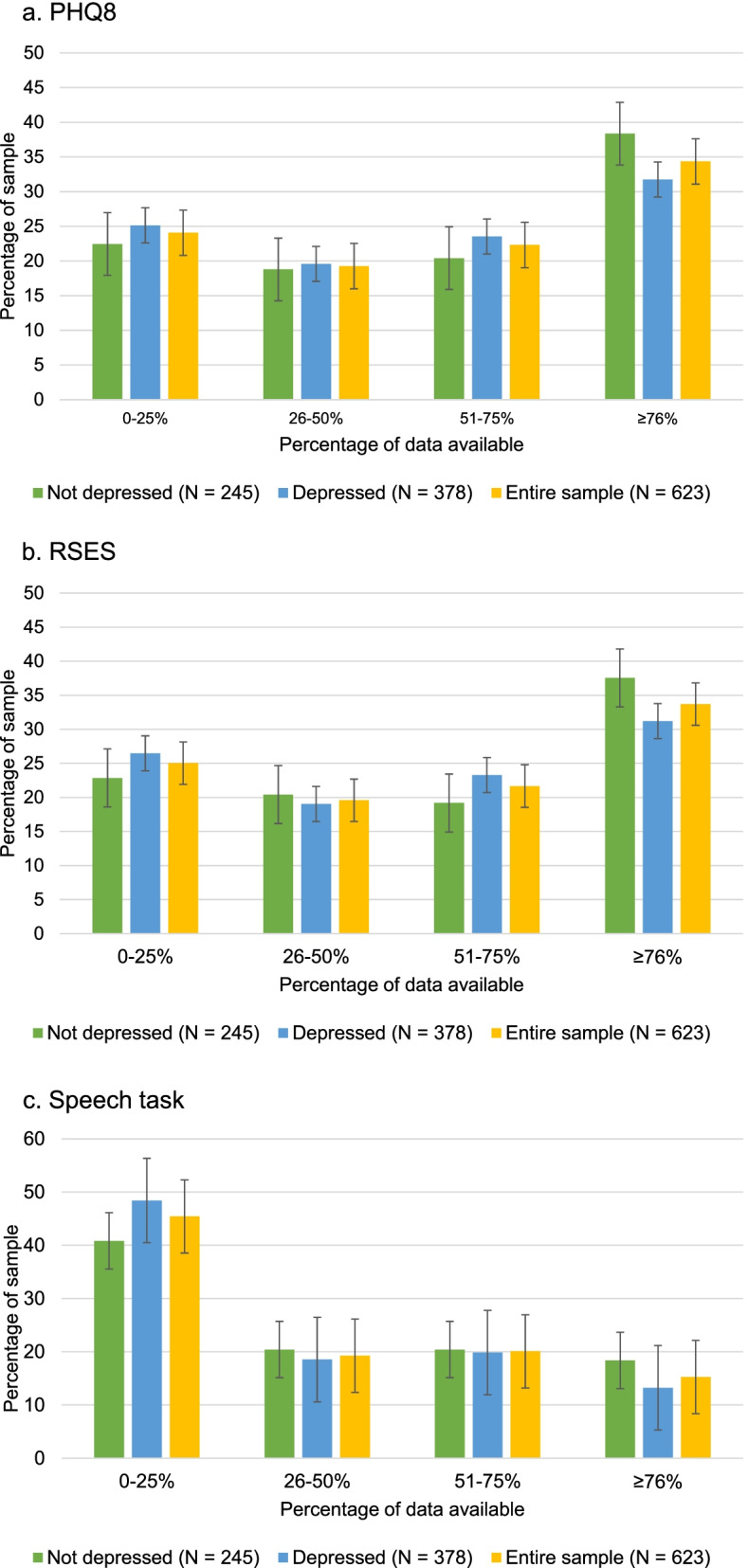
Questionnaires triggered every 2 weeks; maximum number of possible responses: 52. 3a: 8-item Patient Health Questionnaire (PHQ8); 3b: Rosenberg Self-Esteem Scale (RSES); 3c: Speech data

Figure 3a-c display active RMT data collection stratified baseline depression status. Overall, participants completed a median of 21 (IQR:9–31) PHQ-8 questionnaires, 20 (IQR:9–30) RSES questionnaires, 12 (IQR:2–23) speech tasks. A total of 95.3, 94.5 and 82.2% of participants had any data available for the PHQ8, RSES and speech tasks respectively. Chi squared tests found no significant differences in data availability between those with or without depression symptoms at baseline for the PHQ8 (X^2^ (622, *n* = 623) = 3.0, *p* = 0.38), RSES (X^2^ (622, *n* = 623) = 3.83, *p* = 0.28), or speech task (X^2^ = 4.8, *p* = 0.19). The mean numbers of ESM items completed by those with and without depression symptoms at baseline throughout the study duration were 11.8 (SD = 23.7) and 11.9 (SD = 23.7) respectively, with t-tests demonstrating no significant difference in ESM data availability between these groups (*p* = 0.158).

**Fig. 4:**
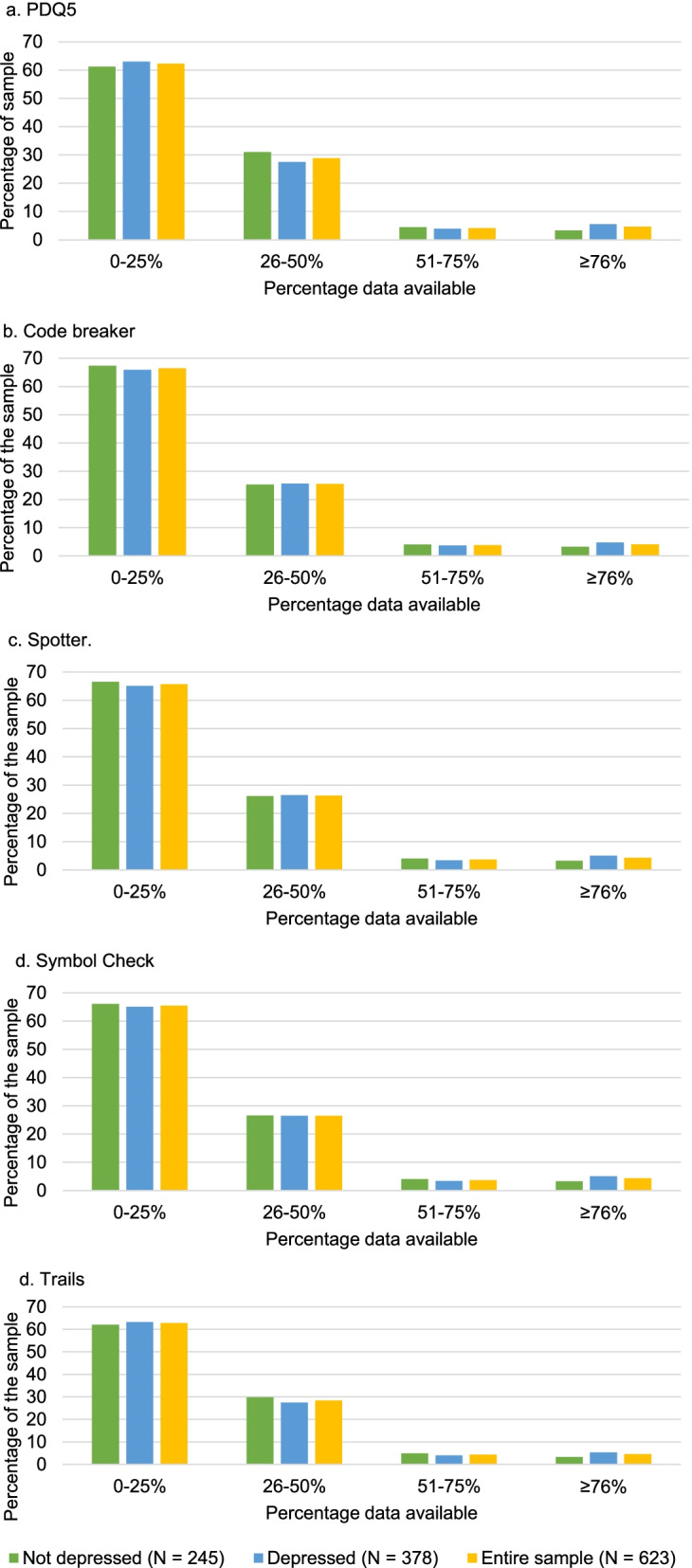
Questionnaires triggered every 6 weeks; maximum number of possible responses: 17. 4a: 5-item Perceived Deficits Questionnaire (PDQ5); 4b: Code Breaker; 4c: Spotter; 4d: Symbol Check; 4e: Trails

Figure 4 displays THINC-it app® data collection stratified baseline depression symptom status. Overall, participants completed a median of 5 (IQR:2–10) THINC-it app® PDQ5 questionnaires, 5 (IQR:2–9) Code Breaker tasks, 5 (IQR:2–9) Spotter tasks, 5 (IQR-2-9) Symbol Check tasks, and 5 (IQR = 2–10) Trails tasks. Over 84% of participants had any data available for the PDQ5 (90.5%), Code Breaker (84.4%), Spotter (84.8%), Symbol Check (84.6%) and Trails (89.9%) tests. Chi squared tests found no significant differences in data availability between those with or without depression at baseline for the PDQ5 (X^2^ (622, *n* = 623) = 2.5, *p* = 0.48), Code Breaker (X^2^ (622, *n* = 623) = 0.91, *p* = 0.82), Spotter (X^2^ (622, *n* = 623) = 1.28, *p* = 0.73), Symbol Check (X^2^ (622, *n* = 623) = 1.26, 0.74) or Trails (X^2^ (622, *n* = 623) = 2.0, *p* = 0.58) tasks.

##### Data collected via wearable technology

**Fig. 5:**
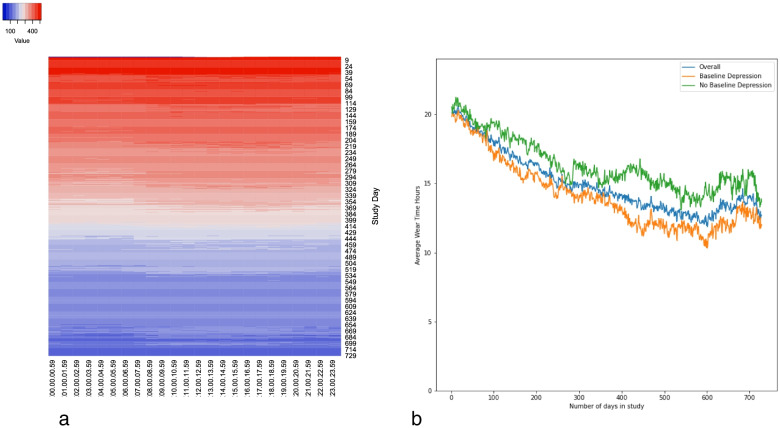
**A**) Heatmap representing study day and data points per hour. **B**) percentage wear time stratified by baseline depression status

Table 2 displays wearable RMT data collection using Fitbit, stratified by baseline depression status. Data collection relied on 1) participants wearing the Fitbit device, 2) regularly charging and syncing the Fitbit device; 3) data being returned/provided by the Fitbit servers. Fitbit wear-time varied during the study (Fig. 5a), with the average participant wear-time across the entire duration of follow-up estimated as 62.5% (SD: 9.1 percentage points, Fig. 5b), and the average number of hours per day as 15.1 h (SD: 2.2 h). Wear-time decreased over time and wear-time did not significantly differ between those with no depression symptoms versus those with symptoms at baseline (X^2^(622, *n* = 623) = 525616, *p*=0.24).

**Table 2:** Wearable remote measurement technology data availability stratified by baseline depression status

Data Type	Data Completion^a^	Total Sample (*n* = 623)	No/Some Baseline Depression Symptoms (*n* = 245)	High Baseline Depression Symptoms (*n* = 378)	*X*^*2*^ *(P* value)
		N (%)	N (%)	N (%)	
Heart Rate	*Any data*	588 (94.4)	229 (93.4)	359 (95.0)	
	*No data*	35 (5.6)	16 (6.5)	19 (5.0)	
	0–25%	103 (16.5)	30 (12.2)	73 (19.3)	5.7 (0.13)
	26–50%	83 (13.3)	32 (13.1)	51 (13.5)	
	51–75%	111 (17.8)	47 (19.2)	64 (16.9)	
	76 + %	326 (52.2)	136 (55.1)	190 (50.6)	
Steps	*Any data*	587 (94.2)	229 (93.4)	358 (94.7)	
	*No data*	36 (6.8)	16 (6.6)	20 (5.3)	
	0–25%	120 (19.3)	38 (15.5)	82 (21.7)	6.5 (0.09)
	26–50%	88 (14.1)	30 (12.2)	58 (15.3)	
	51–75%	116 (18.6)	46 (18.8)	70 (18.5)	
	76 + %	299 (48.0)	131 (53.5)	168 (44.4)	
Sleep (Classic)^b^	*Any data*	543 (87.2)	214 (87.3)	329 (87.0)	
	*No data*	80 (12.8)	31 (12.7)	49 (13.0)	
	0–25%	485 (77.8)	200 (81.6)	285 (75.3)	3.9 (0.28)
	26–50%	98 (15.7)	33 (13.5)	65 (17.2)	
	51–75%	38 (6.1)	11 (4.5)	27 (7.1)	
	76 + %	2 (0.3)	1 (0.4)	1 (0.3)	
Sleep Stages	*Any data*	536 (86.0)	213 (86.9)	323 (85.4)	
	*No data*	87 (14.0)	32 (13.1)	55 (14.6)	
	0–25%	212 (34.0)	80 (32.7)	132 (34.9)	2.3 (0.52)
	26–50%	134 (21.5)	58 (23.7)	76 (20.1)	
	51–75%	152 (24.4)	63 (25.7)	89 (23.5)	
	76 + %	125 (20.1)	44 (18.0)	81 (21.4)	
Activity	*Any data*	580 (93.1)	226 (92.2)	354 (93.7)	
	*No data*	43 (6.9)	19 (7.8)	24 (6.3)	
	0–25%	358 (57.5)	126 (51.4)	232 (61.4)	14.1 (0.002)
	26–50%	143 (23.0)	53 (51.6)	90 (23.8)	
	51–75%	88 (14.1)	48 (19.6)	40 (10.6)	
	76 + %	34 (5.5)	18 (9.4)	16 (4.2)	
Calorie intake^c^	*Any data*	580 (93.1)	224 (91.4)	356 (94.2)	
	*No data*	43 (6.9)	21 (8.6)	22 (5.8)	4.57 (0.21)
	0–25%	122 (19.6)	40 (16.3)	82 (21.7)	
	26–50%	87 (19.6)	31 (12.7)	56 (14.8)	
	51–75%	113 (18.1)	44 (18.0)	69 (18.3)	
	76 + %	301 (48.3)	130 (53.1)	171 (45.2)	

^a^Calculated as the total number of days in which at least one data point has been provided. ^b^Classic sleep data comprise sleep time, restlessness, and awake time. ^c^Data collected either via manual input about food/liquid intake from participant, or Fitbit automation from step count data (not possible to delineate source of data)

Step count data were the most frequently available data, with almost 50% of participants providing > 75% of expected data throughout the course of follow-up. Activity data (comprising a combination of data derived from Fitbit proprietary algorithms and via participants inputting their own activities manually) was the least readily available data, with only 5% of participants having > 75% data availability. Activity data are also the only data type found to have significantly different levels of availability according to the presence of depression at baseline (X^2^ (622, *n* = 623) = 14.1, *p* = 0.002). In comparison to those without depression at baseline, those identified as symptomatic at baseline had a significantly larger percentage of people providing < 26% of activity data. Figure 5a shows a paler horizontal band of colour between days 290 and 380 of study participation, indicating lower levels of wear-time during these time-points, and Fig. 5b shows a dip in percentage wear-time in people with symptoms of depression at baseline after the first year of participation.

##### Data collected via pRMT

Table 3 displays passive data collection across all smartphone sensors, stratified by the presence of baseline depression. The most data were available for GPS location and battery level data. The least data were available for phone usage. No evidence of a difference in data availability between those with and without depression at baseline was identified.

**Table 3:** Passive remote measurement technology data availability stratified by baseline depression status and measurement

Data Type	Data Completion^a^	Total Sample (*n* = 623)	No/Some Baseline Depression Symptoms (*n* = 245)	High Baseline Depression Symptoms (*n* = 378)	*X*^*2*^ *(P* value)
		N (%)	N (%)	N (%)	
Battery Level	*Any data*	603 (96.8)	232 (94.7)	371 (98.1)	
	*No data*	20 (3.2)	13 (5.3)	7 (1.9)	
	0–25%	239 (38.4)	83 (33.9)	156 (41.3)	4.9 (0.18)
	26–50%	126 (20.2)	48 (19.6)	78 (20.6)	
	51–75%	132 (21.2)	57 (23.3)	75 (19.8)	
	76 + %	126 (20.2)	57 (23.3)	69 (18.3)	
Gyroscope	*Any data*	561 (90.0)	217 (88.6)	344 (91.0)	
	*No data*	62 (10.0)	28 (11.4)	34 (9.0)	
	0–25%	293 (47.0)	106 (43.3)	187 (49.5)	4.6 (0.19)
	26–50%	129 (20.7)	48 (19.6)	81 (21.4)	
	51–75%	127 (20.4)	59 (24.1)	68 (18.0)	
	76 + %	74 (11.9)	32 (13.1)	42 (11.1)	
Ambient Light	*Any data*	583 (93.6)	223 (91.0)	360 (95.2)	
	*No data*	40 (6.4)	22 (9.0)	18 (4.8)	
	0–25%	250 (40.1)	89 (36.3)	161 (42.6)	3.7 (0.30)
	26–50%	125 (20.1)	48 (19.6)	77 (20.4)	
	51–75%	122 (19.6)	51 (20.8)	71 (18.8)	
	76 + %	126 (20.2)	57 (23.3)	69 (18.3)	
Ambient Noise	*Any data*	581 (93.3)	222 (90.6)	359 (95.0)	
	*No data*	42 (6.7)	23 (9.4)	19 (5.0)	
	0–25%	273 (43.8)	100 (40.8)	173 (45.8)	5.4 (0.15)
	26–50%	134 (21.5)	47 (19.2)	87 (23.0)	
	51–75%	124 (19.9)	58 (23.7)	66 (17.5)	
	76 + %	92 (14.8)	40 (16.3)	52 (13.8)	
GPS Location	*Any data*	603 (96.8)	232 (94.7)	371 (98.2)	
	*No data*	20 (3.2)	13 (5.3)	7 (1.8)	
	0–25%	246 (39.5)	85 (34.7)	161 (42.6)	5.3 (0.15)
	26–50%	133 (21.3)	51 (20.8)	82 (21.7)	
	51–75%	129 (20.7)	58 (23.7)	71 (18.8)	
	76 + %	115 (18.5)	51 (20.8)	64 (16.9)	
Bluetooth Devices	*Any data*	596 (95.7)	231 (94.3)	365 (96.6)	
	*No data*	27 (4.3)	14 (5.7)	13 (3.4)	
	0–25%	237 (38.0)	82 (33.5)	155 (41.0)	5.4 (0.15)
	26–50%	128 (20.5)	48 (19.6)	80 (21.2)	
	51–75%	133 (21.3)	59 (24.1)	74 (19.6)	
	76 + %	125 (20.1)	56 (22.9)	69 (18.3)	
App Use^b^	*Any data*	579 (92.9)	225 (91.8)	354 (93.7)	
	*No data*	44 (7.1)	20 (8.2)	24 (6.3)	
	0–25%	240 (38.5)	82 (33.5)	158 (41.8)	9.8 (0.02)
	26–50%	133 (21.3)	46 (18.8)	87 (23.0)	
	51–75%	122 (19.6)	58 (23.7)	64 (16.9)	
	76 + %	128 (20.5)	59 (24.1)	69 (18.3)	
Phone interactions^c^	*Any data*	584 (93.7)	225 (9.2)	359 (95.0	
	*No data*	39 (6.3)	20 (8.9)	19 (5.0)	
	0–25%	237 (38.0)	83 (33.9)	154 (40.7)	8.8 (0.03)
	26–50%	136 (21.8)	46 (18.8)	90 (23.8)	
	51–75%	119 (19.1)	55 (22.5)	64 (16.9)	
	76 + %	131 (21.0)	61 (24.9)	70 (18.5)	

^a^Calculated as the total number of days in which at least one data point has been provided. ^b^App names, foreground or background app use, time spent using apps. ^c^How individuals interact with their phones, including phone screen on time, number of interactions with keyboard, screen touches, and extent to which the phone is asleep or awake

## Discussion

### Study recruitment and retention

Recruitment into RADAR-MDD was highly successful, with the flexibility of face-to-face and remote enrolments resulting in the study exceeding its recruitment targets despite the COVID-19 pandemic [42]. Attrition rates in longitudinal research vary widely [43] and whilst there is no recognised threshold for “acceptable” versus “unacceptable” dropout, follow-up levels of 50, 60 and 70% have previously been described as adequate, good and very good respectively [44]. Here we report ~ 80% completion rates of our outcomes across all follow-up timepoints, with 79.8% of all enrolled individuals completing the study protocol for the maximum amount of time possible, representing excellent availability of our primary and secondary outcome measures.

### Sociodemographic characteristics

The RADAR-MDD cohort has a higher proportion of White and female individuals than would typically be seen in the general population or depressed population [45] reflecting the tendency for White females to attend mental health services more often than their male/non-White counterparts, and their greater likelihood of participating in research studies [46, 47]. The mean age and gender distribution in our participants is comparable to other MDD samples, such as Sequenced Treatment Alternatives to Relieve Depression (STAR*D; [48]) and the Rhode Island Methods to Improve Diagnostic Assessment and Services (MIDAS; [49]). These characteristics may limit the generalisability of our findings to the wider population. It is also worth noting that ethnic groups across the two countries who collected ethnicity data are challenging to compare, meaning that in-depth interrogation of racial differences in outcomes will be affected by small cell sizes unless ethnic groups are merged into larger, less descriptive categories. In terms of clinical presentation, our sample have slightly lower levels of current depression severity and reduced WSAS functional disability than those recruited into STAR*D [48].

### RMT domains and data availability

Data availability varied across the RMT domains. Over 90% of participants had data available for analysis from the aRMT, with the PHQ-8 and RSES having the largest amount of data available for the most people. The least amount of aRMT data was available for all assessments conducted via the THINC-it® app, with < 26% of expected data available in approximately 60% of participants. There are several explanations for this difference in data availability in comparison to our other aRMT assessments. Firstly, due to the technical requirements of integrating data from the separate THINC-it® app into the RADAR-base platform, the first THINC-it® data were received in March 2018, with the first 4-months of data collection excluding THINC-it® data. There were also initial challenges syncing data collected via the THINC-it with the RADAR-base platform, meaning there was potential for data loss in the early months of data collection. Secondly, the THINC-it® app is separate to the other RADAR-base apps, with different branding, design and feel to the RADAR-base apps. This may have made the tasks appear separate or “other” to the main protocol and reduced adherence to these tasks. The THINC-it® app does not have an inbuilt notification system - participants received notifications to complete the cognitive tasks via the RADAR-base aRMT app. Participants are required to switch between apps, which increases the number of points at which interest or motivation may be lost [50]. Finally, the cognitive tasks offered as part of the THINC-it requires more attention than conventional questionnaires which may be more challenging for those who are experiencing depression symptoms [51].

We report an overall Fitbit wear-time of 62.5%, across a median study participation of 541 days, and a mean wear-time of 15.1 h per day. This is lower than the wear-time of 22.6 h per day across a two-year follow-up period in a recent United States population-based Fitbit study by Radin and colleagues [52]. However, Radin et al. omitted missing wear time data, and excluded measurements with a wear-time lower than 1000 min per day which inflates their wear-time statistics. In contrast to our sample, Radin et al. [52] used a non-clinical population and the barriers to long-term use of a wearable device are likely to be different in an MDD versus general population sample [21]. Comparatively, Pedrelli et al., 2020 [53] report Empatica E4 wear-time estimates of 92–94% in their study involving 31 individuals with MDD, however their follow-up period was limited to only 8-weeks [53]. Although similar in clinical characteristics, our duration of follow-up and integration of a wearable into a more complex set of data collection sources likely explains the differences in wear time reported.

To the best of our knowledge, no remote measurement studies have reported the quantity of data collected via smartphone sensors. The largest amount of data were available for battery level and GPS sensors. For a multiparametric analysis, data across multiple sensor types will be needed. We report a total of 110 individuals (17.7% of the sample) who have > 50% of data for data types. It is important to acknowledge this as an indicator of the amount of resource and data collection required for multiparametric analyses. Although a remote study by nature, participants had close contact with the research team throughout the study, the researchers were available for technical support and questionnaire reminders, in addition to conducting risk assessments based on questionnaire answers. Future work will need to investigate the minimum amount of contact time required to acquire usable data, for real-world implementation to be viable.

### Limitations

There are several limitations and challenges presented by the current paper. Firstly, each of the sensor and data types collected has different temporal validity and aggregation requirements. For example, sleep data are only meaningful when aggregated from midday-midday, whereas activity data are more relevant when calculated from midnight-midnight. At a granular level, data from smartphone and wearable sensors are so fine that no meaningful inferences can be gained, requiring some form of aggregation which may not be the same across different sensors. For example, whereas heart-rate data might be collected every 5 s and summarised across an hour, the aggregation of GPS data is dependent on the smartphone device being used. In the current paper we have endeavoured to summarise data availability as coherently as possible within these constraints, aiming to provide an easily replicable, comparable, and interpretable description of the data available within our dataset.

It is also essential to acknowledge the technical challenges inherent to multimodal data collection across long periods of time. RADAR-base and its associated apps were developed and piloted within the main data collection period, with iterative changes and updates being made throughout the course of follow-up. These changes may have been implemented to overcome a system-related issue introduced by the updates to the Android operating system, or in direct response to participant or researcher feedback. This flexibility in app design and development is essential to maintain app compatibility. This means that an individual participating throughout 2019–2020 will have had a different user experience to an individual participating throughout 2020–2021.

Whilst the majority of our recruitment occurred before the global pandemic, the threat posed by COVID-19 may have affected existing participants’ research experience and data completion. Recent evidence suggests that people with moderate to severe levels of depression who are already enrolled in a research study show a reduced ability and desire to adhere to research protocols due to COVID-19 [54]. Given the high level of depressive symptoms in our sample, the pandemic and its associated social interventions may have added a burden to participants resulting in an increased dropout rate and reduced adherence to the study protocol. We have previously reported the impact of the pandemic and associated social interventions on the data collected via RMT across the RADAR-CNS clinical studies [55] and future work will extend this to examine how the pandemic may have affected data availability.

Despite these limitations, RADAR-MDD remains the largest, most ambitious multimodal RMT study in depression. A recent systematic review summarising studies using passive and active smartphone-based measurements in affective disorders found only 5 studies in people with MDD, and these studies reported a median sample size of 5, and median follow-up time of 4 weeks, in addition to huge variability in the quality of reporting [17].

### Future research

There are some vital next steps in the exploration of RADAR-MDD data which will be examined in addition to the primary objectives of the RADAR-MDD study [18]. Firstly, as reported earlier, the present paper reports the amount of data available across all modes of data collection. A more thorough investigation into the quality of the data is warranted before more complex analyses are conducted. Furthermore, whilst we show no evidence of a link between baseline depression status and data availability, it is likely that fluctuations in depression symptoms over time are more relevant for predicting technology use, rather than a static baseline status, for example, future work will explore whether missing data due to reduced participant adherence might be an early sign of depressive relapse. We have not described sociodemographic, clinical and technical predictors of data availability which will be the subject of a future paper.

## Conclusion

The data collected in RADAR-MDD indicates that collecting RMT data from clinical populations is feasible. We found comparable levels of data availability in active (requiring input from the participant) and passive (requiring no input from the participant) forms of data collection, demonstrating that both are feasible in this patient group. However, data availability will depend on the data type, with higher burden data sources (such as cognitive tasks, or keeping wearable devices charged) reducing data availability. There was no convincing indication that the severity of depression symptoms at baseline was associated with data availability, in this sample. The next steps are to illustrate the predictive value of these data, which will be the focus of our future data analysis aims.

## Supplementary Information

**Additional file 1.** Schedule of introduction of different RADAR-MDD assessments.

**Additional file 2.** The North Wind and the Sun.

**Additional file 3.** Operationalisation of depression definitions.

**Additional file 4.** Reasons for withdrawal.

**Additional file 5.** Between-site stratification.

## Data Availability

The datasets used and/or analysed during the current study are available from the corresponding author on reasonable request.

## Abbreviations

aRMT: Active Remote Measurement Technology
AUDIT: Alcohol Use Disorders Identification Test
BIPQ: Brief Illness Perceptions Questionnaire
CIBER: Centro de Investigación Biomédica en Red
CIDI-SF: Composite Diagnostic Interview – Short Form
CSRI: Client Service Receipt Inventory
ESM: Experience Sampling Method
GAD7: 7-item Generalised Anxiety Disorder questionnaire
GPS: Global Positioning System
IDS-SR: Inventory of Depressive Symptomatology – Self Report
KCL: King’s College London
LIDAS: Lifetime Depression Assessment – Self-Report
LTE-Q: List of Threatening Experiences Questionnaire
MARS-5: Medication Adherence Report Scale
MDD: Major Depressive Disorder
PDQ: Perceived Deficits Questionnaire
PHQ: Patient Health Questionnaire
PPG: Photoplethysmography
pRMT: Passive Remote Measurement Technology
RADAR-CNS: Remote Assessment of Disease and Relapse – Central Nervous System
RADAR-MDD: Remote Assessment of Disease and Relapse – Major Depressive Disorder
REDCap: Research Electronic Data Capture
RMT: Remote Measurement Technologies
RSES: Rosenberg Self-Esteem Scale
STAR*D: Sequenced Treatment Alternatives to Relieve Depression
VUMC: Amsterdam University Medical Centre
WSAS: Work and Social Adjustment Scale
YLD: Years Lived with Disability
