# Supplementary material for: Remote Assessment of Disease and Relapse in Major Depressive Disorder (RADAR-MDD): recruitment, retention, and data availability in a longitudinal remote measurement study
Source: BMC Psychiatry. 2022 Feb 21;22:136. doi: 10.1186/s12888-022-03753-1 (PMC8860359; doi:10.1186/s12888-022-03753-1)
Supplement: Supplementary file 1 — Additional file 1. Schedule of introduction of different RADAR-MDD assessments. [file 12888_2022_3753_MOESM1_ESM.docx]

## Additional File 1: Schedule of introduction of different RADAR-MDD assessments

| **Site** | **Start of data collection** | **End of data collection** | | **Reason for early removal/late addition** | **Total N days of data collection** | | | |
| --- | --- | --- | --- | --- | --- | --- | --- | --- |
| **London** | 30/11/2017 | 31/03/2021 |  |  | 1271 | | | |
| **Barcelona** | 12/09/2018 | 30/04/2021 |  |  | 961 | | | |
| **Amsterdam** | 12/02/2019 | 31/03/2021 |  |  | 778 | | | |
| **Data type** | **Sensor/assessment** | **Introduced** | **Switched off/ no longer used** |  | **Duration of data collection (days)** | | | |
|  |  |  |  |  | **Total** | **London** | **Barcelona** | **Amsterdam** |
| aRMT | PHQ8 | 30/11/2017 | 31/03/2021 |  | 1271 | 1271 | 931 | 778 |
|  | RSES | 30/11/2017 | 31/03/2021 |  | 1271 | 1271 | 931 | 778 |
|  | THINC-IT | 13/03/2018 | 31/03/2021 |  | 1114 | 1114 | 931 | 778 |
|  | Speech task | 20/08/2018 | 31/03/2021 | Technical – delays in setting up technical infrastructure | 954 | 954 | 931 | 778 |
| Wearable | FitBit Charge 2 | 30/11/2017 | 31/03/2021 |  | 1271 | 1271 | 931 | 778 |
|  | FitBit Charge 3 | 27/02/2019 | 31/03/2021 | Technical – discontinuation of FitBit Charge 2 | 763 | 763 | 763 | 763 |
| pRMT | Acceleration | 30/11/2017 | 24/07/2019 | Feasibility – significant drain on battery life | 601 | 601 | 315 | 162 |
|  | Battery Level | 30/11/2017 | 31/03/2021 |  | 1271 | 1271 | 931 | 778 |
|  | Gyroscope | 30/11/2017 | 31/03/2021 |  | 1271 | 1271 | 931 | 778 |
|  | Ambient Light | 30/11/2017 | 31/03/2021 |  | 1271 | 1271 | 931 | 778 |
|  | Magnetic Field | 30/11/2017 | 24/07/2019 | Feasibility – significant drain on battery life | 601 | 601 | 315 | 162 |
|  | GPS location | 30/11/2017 | 31/03/2021 |  | 1271 | 1271 | 931 | 778 |
|  | Bluetooth devices | 30/11/2017 | 31/03/2021 |  | 1271 | 1271 | 931 | 778 |
|  | Processed Audio | 30/11/2017 | 31/03/2021 |  | 1271 | 1271 | 931 | 778 |
|  | Phone usage | 30/11/2017 | 31/03/2021 |  | 1271 | 1271 | 931 | 778 |
|  | SMS messages | 30/11/2017 | 09/01/2019 | Technical – change in Android data access | 405 | 405 | 119 | 0 |
|  | Call logs | 30/11/2017 | 09/01/2019 | Technical – change in Android data access | 405 | 405 | 119 | 0 |
| RedCap outcome assessments | Depression | 30/11/2017 | 30/04/2021 |  | 1271 | 1271 | 961 | 778 |
|  | Anxiety | 30/11/2017 | 30/04/2021 |  | 1271 | 1271 | 961 | 778 |
|  | Functional Disability | 30/11/2017 | 30/04/2021 |  | 1271 | 1271 | 961 | 778 |
|  | Illness Perceptions | 30/11/2017 | 30/04/2021 |  | 1271 | 1271 | 961 | 778 |
|  | Life events | 30/11/2017 | 30/04/2021 |  | 1271 | 1271 | 961 | 778 |
|  | Service Use | 30/11/2017 | 30/04/2021 |  | 1271 | 1271 | 961 | 778 |
|  | Medication Adherence | 30/11/2017 | 30/04/2021 |  | 1271 | 1271 | 961 | 778 |
|  | Alcohol Use | 30/11/2017 | 30/04/2021 |  | 1271 | 1271 | 961 | 778 |
