# Supplementary material for: Remote Assessment of Disease and Relapse in Major Depressive Disorder (RADAR-MDD): recruitment, retention, and data availability in a longitudinal remote measurement study
Source: BMC Psychiatry. 2022 Feb 21;22:136. doi: 10.1186/s12888-022-03753-1 (PMC8860359; doi:10.1186/s12888-022-03753-1)
Supplement: Supplementary file 2 — Additional file 2. The North Wind and the Sun. [file 12888_2022_3753_MOESM2_ESM.docx]

## Additional file 2: The North Wind and the Sun

**Excerpt 1**

The North Wind and the Sun were disputing which was the stronger, when a traveller came along wrapped in a warm cloak. They agreed that the one who first succeeded in making the traveller take his cloak off should be considered stronger than the other.

**Excerpt 2**

Then the North Wind blew as hard as he could, but the more he blew the more closely did the traveller fold his cloak around him; and at last the North Wind gave up the attempt.

**Excerpt 3**

Then the Sun shone out warmly, and immediately the traveller took off his cloak. And so the North Wind was obliged to confess that the Sun was the stronger of the two.
