# Supplementary material for: Remote Assessment of Disease and Relapse in Major Depressive Disorder (RADAR-MDD): recruitment, retention, and data availability in a longitudinal remote measurement study
Source: BMC Psychiatry. 2022 Feb 21;22:136. doi: 10.1186/s12888-022-03753-1 (PMC8860359; doi:10.1186/s12888-022-03753-1)
Supplement: Supplementary file 3 — Additional file 3. Operationalisation of depression definitions. [file 12888_2022_3753_MOESM3_ESM.docx]

## Additional File 3: Operationalisation of depression definitions

| **Depression definition** | **Measurement Used** | **Definition categories** | **Criteria for membership** |
| --- | --- | --- | --- |
| Symptom status | IDS-SR and CIDI-SF | No symptoms | ≤21 on the IDS-SR and not meeting CIDI-SF criteria for MDD |
|  |  | Some symptoms | ≤25 on the IDS-SR and meeting CIDI-SF criteria for MDD; or >21 on the IDS-SR and not meeting CIDI-SF criteria for MDD. |
|  |  | Symptomatic | ≥26 on IDS-SR and meeting CIDI-SF criteria for MDD. |
| Depressive relapse | IDS-SR and CIDI-SF | No | Having no symptoms or some symptoms; or having shown no change in symptom status. |
|  |  | Yes | Being symptomatic and previously having been in a state of no symptoms.  Switching between these two states within in a 6-month period. |
| Depressive remission | IDS-SR and CIDI-SF | No | Being symptomatic or having some symptoms; or having sown no change in symptom status. |
|  |  | Yes | Having no symptoms and previously having been in a symptomatic state.  Switching between these two extreme states within a 6-month period. |
| Deterioration of symptoms | IDS-SR | No | No significant change in IDS-SR symptom severity in the previous 3 months. |
|  |  | Yes | Showing an increase in IDS-SR score of ≥2SDs since the previous assessment. |
| Improvement of symptoms | IDS-SR | No | No significant change in IDS-SR symptom severity in the previous 3 months. |
|  |  | Yes | Showing a decrease in IDS-SR score of ≤-2SDs since the previous assessment. |
| Symptom severity | IDS-SR | 0-85 | Lower scores indicate low levels of symptom severity; higher scores indicate high levels of symptom severity. |
| Symptom Category (based on validated thresholds) | IDS-SR | None | Scores 0-13 |
|  |  | Mild | Scores 14-25 |
|  |  | Moderate | Scores 26-38 |
|  |  | Severe | Scores 39-48 |
|  |  | Very severe | Scores 49-84 |

IDS-SR Inventory of Depressive Symptomatology – Self Report. CIDI-SF World Health Organisation’s Composite Diagnostic Interview – Short Form. MDD Major Depressive Disorder. SDs Standard Deviations.
