# Supplementary figures and images for: Remote Assessment of Disease and Relapse in Major Depressive Disorder (RADAR-MDD): recruitment, retention, and data availability in a longitudinal remote measurement study
Source: BMC Psychiatry. 2022 Feb 21;22:136. doi: 10.1186/s12888-022-03753-1 (PMC8860359; doi:10.1186/s12888-022-03753-1)

## Additional File 4: Reasons for withdrawal


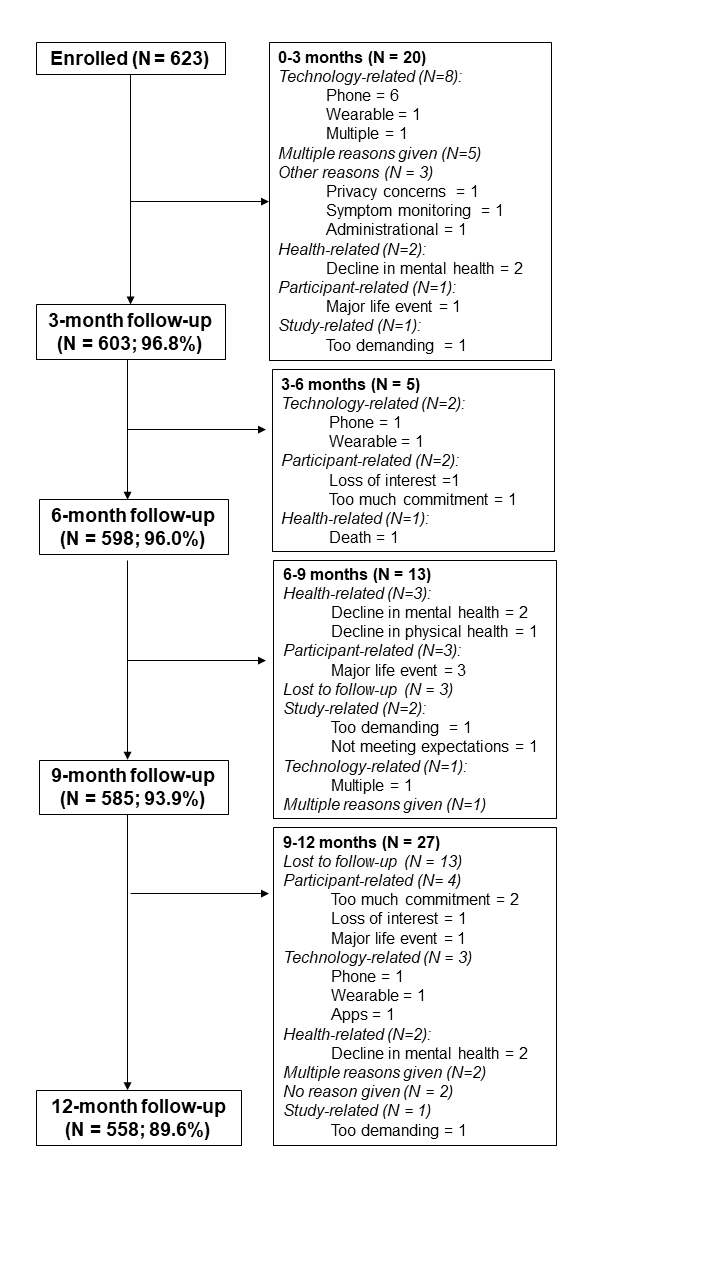


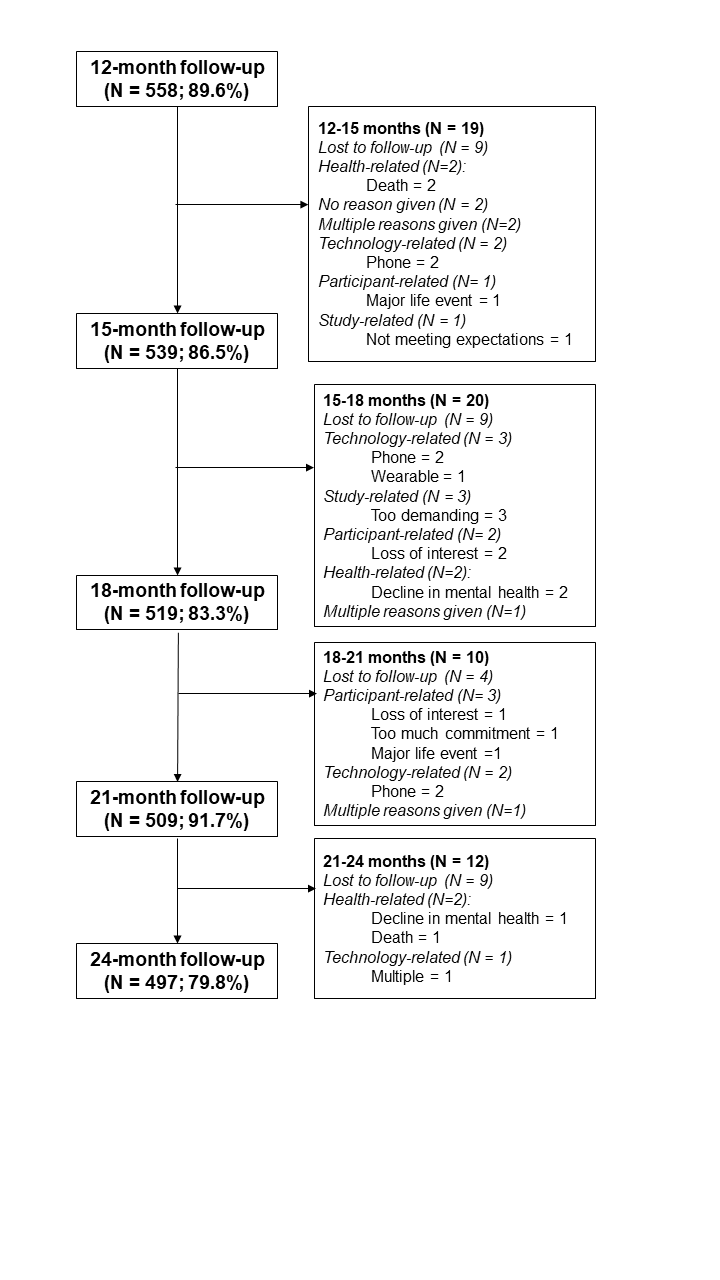

Supplement: Supplementary file 4 — Additional file 4. Reasons for withdrawal. [file 12888_2022_3753_MOESM4_ESM.docx]
