# Supplementary material for: Remote Assessment of Disease and Relapse in Major Depressive Disorder (RADAR-MDD): recruitment, retention, and data availability in a longitudinal remote measurement study
Source: BMC Psychiatry. 2022 Feb 21;22:136. doi: 10.1186/s12888-022-03753-1 (PMC8860359; doi:10.1186/s12888-022-03753-1)
Supplement: Supplementary file 5 — Additional file 5. Between-site stratification. [file 12888_2022_3753_MOESM5_ESM.docx]

## Additional File 5: between-site stratification

|  |  | **Total Sample** | **London** | **Barcelona** | **Amsterdam** | **p-value^1^** |
| --- | --- | --- | --- | --- | --- | --- |
| Total, N(%) |  | 623 (100.0) | 350 (56.2) | 155 (24.9) | 118 (18.9) |  |
| No symptoms |  | 10 (1.6) | 4 (1.1) | 3 (1.9) | 3 (2.5) | 0.012 |
| Some symptoms |  | 237 (38.0) | 147 (42.0) | 41 (26.5) | 49 (41.5) |  |
| Symptomatic |  | 376 (60.4) | 199 (56.9) | 111 (71.6) | 66 (55.9) |  |
| *Socio-demographics* |  |  |  |  |  |  |
| Age, M(SD) |  | 46.4 (15.3) | 44.3 (15.3) | 54.0 (10.7)*** | 42.5 (16.9) |  |
| Gender, N(%) | Female | 471 (75.6) | 267 (74.0) | 112 (72.3) | 92 (78.0) |  |
| Marital Status, N(%) | Single | 223 (35.8) | 136 (38.9) | 26 (16.7) | 61 (51.7) | <0.0001 |
|  | Cohabiting/LTR | 95 (15.3) | 62 (17.7) | 14 (9.0) | 19 (16.1) |  |
|  | Married | 196 (31.5) | 103 (29.4) | 71 (45.8) | 22 (18.6) |  |
|  | Separated | 19 (3.1) | 12 (3.4) | 7 (4.5) | 0 (0.0) |  |
|  | Divorced | 69 (11.1) | 28 (8.0) | 26 (16.8) | 15 (12.7) |  |
|  | Widowed | 21 (3.4) | 9 (2.6) | 11 (7.1) | 1 (0.9) |  |
| Ethnicity, N(%) | White British | 260 (41.7) | 260 (74.3) | - | 0 (0.0) | <0.0001 |
|  | White Dutch | 109 (17.5) | 0 (0.0) | - | 109 (92.4) |  |
|  | White Other | 35 (5.6) | 35 (10.0) | - | 0 (0.0) |  |
|  | African | 2 (0.3) | 2 (0.6) | - | 0 (0.0) |  |
|  | Caribbean | 8 (1.3) | 8 (2.3) | - | 0 (0.0) |  |
|  | Other/Mixed Black | 8 (1.3) | 8 (2.3) | - | 0 (0.0) |  |
|  | Surinamese | 3 (0.5) | 0 (0.0) | - | 3 (2.5) |  |
|  | Indian/Pakistani/Bangladeshi | 13 (2.1) | 6 (1.7) | - | 0 (0.0) |  |
|  | Other/Mixed Asian | 8 (1.3) | 8 (2.3) | - | 0 (0.0) |  |
|  | Other | 22 (3.5) | 16 (4.6) | - | 6 (5.1) |  |
|  | Not collected | 155 (24.9) | 0 (0.0) | 155 (100.0) | 0 (0.0) |  |
| Employment Status | Employed | 257 (41.3) | 183 (52.3) | 35 (22.6) | 39 (33.1) | <0.0001 |
|  | Unemployed | 68 (10.9) | 37 (10.6) | 23 (14.8) | 8 (6.8) |  |
|  | Off sick | 66 (10.6) | 19 (5.4) | 24 (15.5) | 23 (19.5) |  |
|  | Student | 68 (10.9) | 35 (10.0) | 6 (3.9) | 27 (22.9) |  |
|  | Retired | 123 (19.7) | 56 (16.0) | 56 (36.1) | 11 (9.3) |  |
|  | Housewife/Househusband | 22 (3.5) | 7 (2.0) | 10 (6.5) | 5 (4.2) |  |
|  | Furloughed | 3 (0.5) | 3 (0.9) | 0 (0.0) | 0 (0.0) |  |
|  | Carer/Volunteer | 12 (1.9) | 7 (2.0) | 0 (0.0) | 5 (4.2) |  |
|  | Not reported | 4 (0.6) | 3 (0.9) | 1 (0.7) | 0 (0.0) |  |
| Total years in education, M(SD) |  | 16.4 (6.5) | 17.2 (5.3) | 12.5 (4.9)*** | 19.2 (8.8)** |  |
| Receipt of any benefits, N(%) | Yes | 275 (44.1) | 232 (66.3) | 51 (32.9) | 65 (55.1) | <0.0001 |
| Accommodation type, N(%) | Own outright | 167 (26.8) | 82 (23.4) | 74 (47.7) | 11 (9.3) | <0.0001 |
|  | Own with mortgage | 152 (24.4) | 76 (21.7) | 38 (24.5) | 38 (32.2) |  |
|  | Private rental | 124 (19.9) | 107 (30.6) | 10 (6.5) | 7 (5.3) |  |
|  | Local authority rental | 141 (22.6) | 44 (12.6) | 26 (16.8) | 22 (18.6) |  |
|  | Living rent-free | 38 (6.1) | 29 (8.3) | 0 (0.0) | 0 (0.0) |  |
|  | Not reported | 1 (0.2) | 1 (0.3) | 0 (0.0) | 0 (0.0) |  |
| *Clinical Characteristics* |  |  |  |  |  |  |
| Current smoker, N(%) | Yes | 126 (20.2) | 47 (13.4) | 62 (40.0) | 17 (14.4) | <0.0001 |
| Medical comorbidity, N(%) | Yes | 343 (55.1) | 206 (58.9) | 98 (63.2) | 39 (33.1) | <0.0001 |
| Personality Disorder, N(%) | Yes | 480 (77.1) | 268 (76.6) | 133 (85.8) | 79 (67.0) | 0.001 |
| Lifetime traumatic events, N(%) | None | 66 (10.6) | 42 (12.0) | 15 (9.7) | 9 (7.6) | 0.066 |
|  | 1-5 | 360 (57.8) | 205 (58.6) | 85 (54.8) | 70 (59.3) |  |
|  | 6-12 | 185 (29.7) | 92 (26.3) | 55 (35.5) | 38 (32.2) |  |
|  | Not reported | 12 (1.9) | 11 (3.1) | 0 (0.0) | 1 (0.9) |  |
| Current depression | IDS-SR total, M(SD) | 31.3 (14.5) | 29.4 (13.6) | 38.4 (15.3)*** | 28.0 (12.7) |  |
|  | None (0-13), N(%) | 61 (10.1) | 38 (10.9) | 7 (4.5) | 16 (13.6) | <0.0001 |
|  | Mild (14-23), N(%) | 157 (25.9) | 97 (27.7) | 24 (15.5) | 36 (30.5) |  |
|  | Moderate (24-36), N(%) | 206 (33.9) | 124 (35.4) | 40 (25.8) | 42 (35.6) |  |
|  | Severe (37-46), N(%) | 104 (17.1) | 51 (14.6) | 38 (24.5) | 15 (12.7) |  |
|  | Very severe (47-84), N(%) | 79 (13.0) | 30 (8.6) | 40 (25.8) | 9 (7.6) |  |
|  | Not reported | 16 (2.6) | 10 (2.9) | 6 (3.9) | 0 (0.0) |  |
| Suicidal ideation, N(%) | Yes | 110 (17.7) | 48 (13.7) | 36 (23.2) | 26 (22.0) | 0.05 |
| Taking antidepressants, N(%) | Yes | 408 (65.5) | 206 (58.9) | 141 (91.0) | 61 (51.7) | <0.001 |
| Current anxiety | GAD7 total, M(SD) | 8.8 (5.7) | 8.0 (5.4) | 11.6 (5.1)*** | 7.6 (4.8) |  |
|  | None (0-5), N(%) | 200 (32.1) | 136 (38.9) | 21 (13.6) | 43 (36.4) | <0.001 |
|  | Mild (6-10), N(%) | 153 (24.6) | 86 (24.6) | 29 (18.7) | 38 (32.2) |  |
|  | Moderate (11-15), N(%) | 152 (24.4) | 73 (20.9) | 53 (34.2) | 26 (22.0) |  |
|  | Severe (16-21), N(%) | 118 (18.9) | 55 (15.7) | 52 (33.6) | 11 (9.3) |  |
| Current functional disability | WSAS total, M(SD) | 19.3 (11.1) | 17.2 (10.5) | 23.4 (12.3)*** | 20.0 (9.4)* |  |
|  | No impairment (0-10), N(%) | 155 (24.9) | 104 (29.7) | 28 (18.1) | 23 (19.5)*** | <0.001 |
|  | Some impairment (11-20), N(%) | 154 (24.7) | 100 (28.6) | 25 (16.1) | 29 (24.6) |  |
|  | Significant impairment (>20), N(%) | 314 (50.4) | 146 (41.7) | 102 (65.8) | 66 (55.9) |  |
| Alcohol use | AUDIT total, M(SD) | 3.2 (4.4) | 4.2 (5.0) | 0.8 (1.5)*** | 3.4 (4.1) |  |
|  | Low risk (0-7), N(%) | 528 (84.8) | 281 (80.3) | 150 (96.8) | 97 (82.2) | <0.001 |
|  | Medium risk (8-15), N(%) | 52 (8.4) | 37 (10.6) | 1 (0.7) | 14 (11.9) |  |
|  | High risk (16-19), N(%) | 10 (1.6) | 8 (2.3) | 0 (0.0) | 2 (1.7) |  |
|  | Addiction likely (>19), N(%) | 8 (1.3) | 7 (2.0) | 0 (0.0) | 1 (0.9) |  |
|  | Not reported | 25 (4.0) | 17 (4.9) | 4 (2.6) | 4 (3.4) |  |
| Illness Perceptions, M(SD) | Consequences | 6.1 (2.8) | 5.7 (2.7) | 7.1 (2.8)*** | 6.1 (2.9) |  |
|  | Timeline | 7.1 (3.1) | 7.3 (3.0) | 7.7 (2.8) | 5.6 (3.2)*** |  |
|  | Personal Control | 4.2 (2.7) | 4.3 (2.5) | 3.7 (3.1)* | 4.6 (2.5) |  |
|  | Treatment Control | 6.0 (2.8) | 5.8 (2.8) | 6.4 (2.9) | 5.8 (2.4) |  |
|  | Identity | 5.9 (2.5) | 5.5 (2.4) | 6.9 (2.3)*** | 5.6 (2.5) |  |
|  | Concern | 6.3 (2.9) | 5.7 (2.7) | 8.4 (2.4)*** | 5.2 (2.7)* |  |
|  | Understanding | 6.8 (2.8) | 6.9 (2.5) | 7.2 (3.4) | 5.0 (2.8)** |  |
|  | Emotional Response | 7.1 (2.5) | 6.9 (2.4) | 8.4 (2.2)*** | 6.4 (2.6)* |  |
| aRMT depression (N=598) | PHQ8 total, M(SD) | 10.9 (6.1) | 9.8 (5.7) | 14.1 (6.1) | 9.6 (5.4) | <0.0001 |
|  | None (0-4), N(%) | 98 (16.4) | 61 (17.4) | 13 (8.4) | 21 (17.8) | <0.0001 |
|  | Mild (5-9), N(%) | 171 (28.6) | 107 (30.6) | 23 (14.8) | 41 (34.8) |  |
|  | Moderate (10-14), N(%) | 154 (25.8) | 90 (25.7) | 37 (23.9) | 27 (22.9) |  |
|  | Severe (15-19), N(%) | 109 (18.2) | 41 (11.7) | 44 (28.4) | 24 (20.3) |  |
|  | Extreme (>19), N(%) | 66 (11.0) | 26 (7.4) | 35 (22.6) | 4 (3.4) |  |
| aRMT self-esteem (N=593) | RSES total, M(SD) | 16.8 (2.6) | 17.0 (2.1) | 16.8 (3.0) | 16.6 (2.1) | 0.525 |

^1^p-value calculated via chi-squared across groups. *p<0.05 in linear regression. **p<0.01 in linear regression. ***p<0.001 in linear regression. . IDS-SR Inventory of Depressive Symptomatology – Self Report. GAD7 7-item questionnaire for Generalised Anxiety Disorder. WSAS Work and Social Adjustment Scale. AUDIT Alcohol Use Disorders Identification Test. BIPQ Brief Illness Perceptions Questionnaire. M(SD) Mean (Standard Deviation)
